# Canine Distemper Virus in Mexico: A Risk Factor for Wildlife

**DOI:** 10.3390/v17060813

**Published:** 2025-06-03

**Authors:** Juan Macías-González, Rebeca Granado-Gil, Lizbeth Mendoza-González, Cesar Pedroza-Roldán, Rogelio Alonso-Morales, Mauricio Realpe-Quintero

**Affiliations:** 1 Departamento de Medicina Veterinaria, Hospital Veterinario de Pequeñas Especies, Centro Universitario de Ciencias Biológicas y Agropecuarias, Universidad de Guadalajara, Jalisco 45200, Mexico; juanm.maciasg@academicos.udg.mx (J.M.-G.);; 2 Laboratorio de Investigación en Ciencias Animales, Centro Universitario de Ciencias Biológicas y Agropecuarias, Universidad de Guadalajara, Jalisco 45200, Mexico; 3 Laboratorio de Inmunología y Genética Molecular, Centro Universitario de Ciencias Biológicas y Agropecuarias, Universidad de Guadalajara, Jalisco 45200, Mexico; cesar.pedroza@academicos.udg.mx; 4 Facultad de Medicina Veterinaria y Zootecnia, Universidad Nacional Autónoma de México, Ciudad de México 04510, Mexico; ralonsom@unam.mx

**Keywords:** canine distemper virus, morbillivirus, wildlife, reservoir species, zoonotic risk

## Abstract

Canine distemper is caused by a morbillivirus similar to others that affect livestock and humans. The increase in host range and its persistence in wildlife reservoirs complicate eradication considerably. Canine distemper virus has been reported in wildlife in Mexico since 2007. Dogs were previously considered the main reservoirs, but high vaccination coverage in the USA has helped control the disease, and racoons (*Procyon lotor*) are now recognized as the main reservoirs of the agent in the USA, since they live in high densities in urban environments (peridomestic), where contact with domestic and wildlife species is common. Racoons are now considered to spread CDV in wildlife species and zoo animals. Mexico is home to at least two wildlife species that have been reported as carriers of the CDV infection in studies in the USA. Raccoons and Coyotes are distributed in several Mexican states and could play the same reservoir role as for the US. In addition, the increase in non-traditional pets expands the availability of susceptible individuals to preserve CDV in domiciliary and peri-domiciliary environments, contributing to the spread of the disease. Combined with incomplete vaccination coverage in domestic canids, this could contribute to maintaining subclinical infections. Infected pets with incomplete vaccination schedules could also spread CDV to other canines or wildlife coexisting species. In controlled habitats, such as flora and fauna sanctuaries, protected habitats, zoo collections, etc., populations of wildlife species and stray dogs facilitate the spread of CDV infection, causing the spilling over of this infectious agent. Restricting domestic pets from wildlife habitats reduces the chance of spreading the infection. Regular epidemiological surveillance and specific wildlife conservation practices can contribute to managing threatened species susceptible to diseases like CDV. This may also facilitate timely interventions in companion animals which eventually minimize the impact of this disease in both scenarios. Aim: The review discusses the circulation of CDV in wildlife populations, and highlights the need for epidemiological surveillance in wildlife, particularly in endangered wildlife species from Mexico. Through an extensive review of recent scientific literature about CDV disease in wildlife that has been published in local and international databases, the findings were connected with the current needs of information from a local to global perspective, and conclusions were made to broaden the context of Mexican epidemiological scenarios as closely related to the neighboring regions.

## 1. Introduction

Canine distemper virus (CDV) belongs to the family Paramyxoviridae, genus Morbillivirus. This genus includes agents that produce multisystemic diseases with high morbidity and mortality rates. Their clinical presentations are similar, ranging from subclinical to lethal, characteristic signs including fever, respiratory, and/or gastrointestinal signs. CDV and measles virus (MeV) can also damage the integumentary and nervous systems, causing complications that affect host survival [1,2,3].

It is hypothesized that the disease produced by CDV derived from old variants of MeV or ancestral agents which were transmitted through spillover from humans to dogs in the domestication period of pets. This is based on the first description of a similar disease in dogs from South America in 1753, following the occurrence of measles that was spread by the arrival of Europeans during the colonization of the continent. This theory is supported by the genetic-molecular characteristics shared by both agents, and the similarities between the clinical signs [2,4].

## 2. Morbilliviruses Cause Diseases in Different Species

Unlike MeV, which only infects humans and non-human primates, CDV has the ability to infect a wide and growing host range, including domestic canids and other species of the order Carnivora. The increase in wildlife hosts and their persistence makes eradication of the disease impossible. Species considered reservoir hosts maintain active infection among wild populations allowing the circulation of variants, complicating the epidemiological scenario with other animals, and contributing to the adaptation and further evolution of this infectious agent with the concomitant risk for future threats to animal and human populations [2,5,6].

The disease caused by CDV has a worldwide distribution, its persistence may be due to multiple factors and gives rise to severe outbreaks in vaccinated dogs occurring with increasing frequency. The effectiveness of attenuated viruses used as vaccine strains, as the default management for this disease in domestic dogs, is a common concern based on the increasing antigenic differences between vaccine strains and circulating field variants [7,8,9]. Prevention strategies using vaccines are not widely used in other species [10].

### Genetic Basis of Morbillivirus Diversity

CDV is a single-stranded RNA virus of negative polarity. Its genome consists of six genes encoding the proteins of the virion, nucleoprotein, viral polymerase (large), phosphoprotein, matrix protein and the two surface glycoproteins, fusion protein, and hemagglutinin (H). The H protein is responsible for virus–host cell binding and is the most variable antigen described in members of the genus *Morbillivirus*, which is also used to classify the genetic variants of this agent worldwide [4,11,12,13].

Protruding from the membrane surface of morbilliviruses, the viral hemagglutinin protein mediates binding to cellular receptors; it also plays a key role in determining tissue tropism, the host range of CDV, and is the main target for immune response in the host. The high variability of the h gene is utilized for genetic analysis and for studying the antigenic diversity of CDV. An amino acidic variation of up to 10% between field variants and vaccine strains has been reported by molecular–genetic analysis, which could be related to changes in antigenicity and eventually could affect the variable degree of protection provided by vaccines [5,11,14,15].

In addition to providing a basis for the classification of lineages through the analysis of the h gene, viral variants have been described with specific polymorphisms associated with their origin whether from domestic or wildlife based on specific changes in amino acid residue 549. The identification of Tyrosine (Y) in this position is related to an isolation from a domestic dog, while the appearance of histidine (h) determines that it comes from wild canids. However, there have been exceptions to this determination. This substitution identification tool is also associated with high virulence in variants affecting wildlife [4,13,16].

Another important region of hemagglutinin consists of amino acidic residues 364–392. By the extrapolation of data, it has been determined that the immunodominant epitope of morbilliviruses is located in these positions, that is, a region that mainly attracts the immune response of the host. Studies based on the analysis of this region have made it possible to identify the divergence between vaccine strains and variants obtained in different species. An interesting finding resulting from this kind of research allowed highlighting raccoons as a species that plays an important role in the evolution of the agent, since very different variants have been obtained from them compared to those of other wild animals, in addition to the possibilities that recombination may occur through their infection [5,6].

## 3. Wildlife Hosts of CDV Infection

CDV mainly infects members of the order Carnivora; however, infection is also reported in families of the orders Rodentia, Primates, Artiodactyla, and Proboscidea, occurring in wildlife worldwide [3].

**Figure 1:**
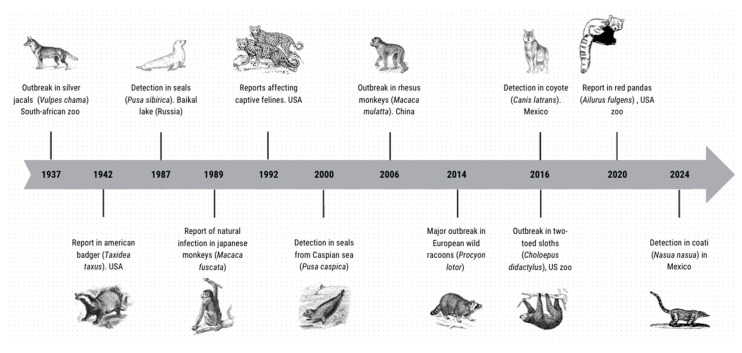
Timeline indicating some non-domestic species as hosts of CDV. Based on the published reports [3,4,17,18,19,20,21].

Until 2016, it was estimated that the country with the highest reports of CDV in wildlife animals was the United States, followed by Japan and Canada, while other countries have few reports on the detection and characterization of the agent in non-domestic species [3,4,17,18]. Since 1937 when a zoo in South Africa provided the first report in non-domestic wildlife from an outbreak in silver jackals (*Vulpes chama*), until 2006 in China where a large population of rhesus monkeys (*Macaca mulatta*) were affected [19,20], countless descriptions of CDV circulation in wildlife have been accumulated globally, as illustrated in Figure 1.

Subsequently, in 1942, the first case of CDV in the American badger (*Taxidea taxus*) was reported in Colorado, USA. In 1988 it was reported in seals from Lake Baikal and the Caspian Sea, while in Arizona it was reported in collared peccaries (*Tayassu tajacu*). The first cases reported in felids occurred in Switzerland based on a retrospective analysis of samples of zoological collections, which confirmed the presence of CDV antigen in tissues from 1972. Subsequently, in the United States, it was reported in captive exotic felids between 1991 and 1992. In 1994, an outbreak was reported in felids residing in the Serengeti National Parks in Tanzania. In addition, there have been reports in non-human primates such as the Japanese macaque (*Macaca fuscata*), naturally infected for the first time in 1989 in Japan, and in 2006 in China in rhesus monkeys (*Macaca mulatta*) where a large population was infected [19,20,22], generating concern for their possible zoonotic potential [2,4,18].

### 3.1. Antecedents in Mammals

Besides a wide variety of terrestrial host species, the genus Morbillivirus is considered important in the conservation of aquatic mammals due to the high mortality rates that have been reported in their populations. Phocine Distemper Virus (PDV), Cetacean Morbillivirus (CMV), and CDV produce damage to the lymphoid and epithelial systems, potentially causing neurological disease, similar to the clinical presentations shown by terrestrial animals infected with CDV. The macroscopic and microscopic lesions caused by the three agents identified in aquatic mammals are similar [23].

The first reports of important epidemics in aquatic mammals caused by CDV variants of terrestrial origin were reported in the late 1980s, the most notable being those in Lake Baikal seals (*Pusa sibirica*) in 1987 and in 2000 in seals in the Caspian Sea (*Phoca capsica*). In 1988, an outbreak was reported in common seals (*Phoca vitulina*) in northern Europe, from which it was identified that the causal agent of this epizootic was a new morbillivirus called PDV. Later on, morbilliviruses other than CDV were identified as agents responsible for massive fatal outbreaks in seals and cetaceans that occurred between 1980 and 1990 [19,23,24,25].

Based on the identification of these agents in aquatic fauna, it has been hypothesized that during a small ice age in the 17th century, the increase in ice cover and low temperatures favored contact between aquatic and terrestrial mammal species, where adaptation in the terrestrial CDV may have occurred. Another remarkable fact that is taken into account for the formulation of hypotheses about its adaptation in both types of fauna is that seal carcasses have been fed to sled dogs for centuries [25]. Currently, CDV infection in pinnipeds is associated mainly with terrestrial origin transmission from the aquatic–terrestrial interface where marine mammals have contact with respiratory exudates, secretions, and excretions with infecting particles from terrestrial mammals [19,23,26] and this could also favor adaptation in both types of fauna.

### 3.2. Reservoir Species

CDV is labile in the environment, so it depends on susceptible hosts (reservoirs) to persist in ecosystems [6,17]. To consider a species as a reservoir, it must be concentrated in large populations that allow it to maintain its enzootic state, i.e., the disease is maintained in the same geographical location for a long time. However, since the vaccination of domestic dogs has reduced the disease in countries with good coverage, the boreal raccoon (*Procyon lotor*) is now recognized as the main reservoir due to reports indicating that the interaction of infected raccoons allowed the spread of CDV to other species in the wild, and to animals in zoological collections. As a species that usually reaches high densities in urban environments (peridomestic), where the interaction of domestic and wild fauna is facilitated, it fulfills the necessary characteristics to maintain the circulation of CDV viral variants in the vicinity of human settlements [4,5,6,27].

Recently, based on a broad review of different hosts of CDV, main reservoirs were defined as corresponding to the carnivorous class; meanwhile, a secondary class was proposed for other non-carnivorous animals, where CDV infection has been reported. Analyses confirmed host-generalist capacity and diversity in CDV, still having dogs as the prevalent species, and red fox (*Vulpes vulpes*) as the principal host in the wild [3]. Raccoons associated with urban areas of Canada have tested positive for CDV, as well as grey foxes in North America, where many studies have been conducted recently [4].

In North America, besides the raccoon, the coyote (*Canis latrans*) is recognized as another important reservoir species, since it also has the ability to spread the agent in peri-urban environments affecting wildlife and domestic animals [28]. Although both reservoir species occur in Mexico, and no studies involve detecting the agent in these species, their importance should still be considered for the local epidemiology of the disease [29].

There are few reports of evidence of CDV infection in Mexican wildlife (see Table 1). Most of the studies have been from monitoring together several pathologies that can affect free-living animals, and some serology-based reports have complemented these with a detection of the virus, which extends the findings obtained in other regions of the world.

### 3.3. Comparative Clinical Signs in Non-Domestic Species

The pathogenesis has not been consistently described in wildlife hosts in which transmission usually occurs by direct contact during predation, fighting, and mating [4,33].

Although the pathogenesis in different host species is very similar, the clinical presentation ranges from subclinical to death, depending on the species, the animal’s immune response, and the viral variant. Due to its tropism for epithelial tissue, CDV produces respiratory and gastrointestinal clinical signs in most hosts [4]. Comparative clinical signs between species have a reported similarity between domestic dogs, wild canids, and mustelids, and other species-dependent signs such as fever, oculonasal discharge, vomiting, diarrhea, anorexia, depression, digital hyperkeratosis, and damage to central nervous system, which can occur showing differences mainly due to the specific pathogenesis in each host [6,33].

In cases of large felids, abnormal behaviors associated with CDV infection causing neuronal disease are reported. Endangered species such as Amur tigers exhibited prominent clinical manifestation behaviors like disorientation, lack of response to stimuli, and/or non-aggressive depression, and CDV was identified as the causal agent. In Russia, low CDV vaccination coverage was found in domestic dogs, and the eventual route of transmission was demonstrated since they were preys of those tigers in the region [34].

Felids in the genus Panthera show gastrointestinal, respiratory, and nervous signs. Altered behavioral states are reported in tigers and lions, as well as convulsions and myoclonus similar to those observed in canids. However, only mild signs are reported in species such as pumas (Puma concolor), ocelots (*Leopardus pardalis*), servals (*Leptailurus serval*), and margays (*Leopardus wiedii*), with only gastrointestinal and respiratory signs described. Occasionally in these species, subclinical presentation is reported, resulting seropositive [4,33].

## 4. Risk to Species Conservation

Since CDV infects a wide variety of carnivores, CDV should be considered an important pathogen in wildlife conservation programs and species extinction risk assessment. Currently CDV is considered a generalist pathogen, so epidemiological assessment of the importance of this disease for populations of wild carnivore species is required. Similar pathogens like the rabies virus, are well known for having reservoir wildlife species and for transmitting disease between domestic animals and endemic fauna [13,29]. This situation is increasingly documented for CDV which is now described as affecting more animal species than rabies virus [17].

The conservation statuses (IUCN; www.iucnredlist.org, accessed on 10 April 2025) for some carnivorous animal species distributed in Mexico are not systematically listed; also, diseases causing a significant reduction in their natural populations are not included either. However, species such as big cats, the Cozumel raccoon (*Procyon pygmaeus*), and the black-footed ferret (*Mustela nigripes*), which have been reported as susceptible to CDV and similar diseases, have been reported in Mexico [4]. Although multiple mammalian families have been reported to be affected by CDV, the majority of cases include the *Canidae*, *Felidae*, and *Mustelidae* families. Several IUCN-listed threatened species belong to these families. In addition to IUCN listings, special attention must be paid to the classification to CDV hosts as the carnivorous class or not-carnivorous class [3]. This has important implications for the distinction between risk and conservation status, and could better reflect the epidemiological frame for Distemper Disease in the wild.

In North America, the significant decline in black-footed ferret populations was considered to be due to bubonic plague and CDV infection [4,35]. Subsequent reintroductions of restored populations of this species to normal levels in their original habitat were made [35]. In 2005, pygmy raccoon populations were declining, and for this reason a study was conducted to identify possible etiological agents that could be causing fatal diseases in the species, with CDV being one of these [36]. However, the detection of the agent in this species was confirmed only two years later using RT-PCR in the results of another study [30]. In ferrets, the impact of CDV on their natural populations is well documented, and it could be considered as representative of the scenario affecting other animal species.

Although more livestock and increased populations of traditional pets close to wildlife habitats have increased the probability of spillover between them [13], few studies have evaluated the edge effect that could explain disease spread between adjacent habitats [2].

### 4.1. Risk of Contagion and Disease in Non-Conventional Pets

Unconventional pets (ferrets, raccoons, coatis, etc.) are becoming more common and are known to host CDV. Since there are no accepted vaccination schedules, there is little information on disease prevention, and available therapeutics are only validated in domestic species; thus, such pets can be associated with spreading diseases such as CDV. The conservation status of some unusual pets is unknown, and the risk of contagion for pets with subclinical disease complicates epidemiological prevention and control.

Ferrets are highly susceptible to CDV, with mortality rates reaching 100% [4,37]. In Mexico, coatis or badgers (*Nasua narica*) have been identified to be infected by CDV based on serologic tests (Table 1) and on direct CDV detection [29].

### 4.2. Prevention Strategies Considering Wildlife Species

The only tool to prevent diseases caused by morbilliviruses is vaccination [4]; however, in most wildlife species, there is no well-defined vaccination schedule as in domestic canids. Therefore, case-by-case analyses are required to establish guidelines and directives oriented to control the spread of these diseases to wildlife.

In several studies, through a comparative genetic analysis between sequences obtained in domestic and wild animals, interspecies contagions have been evidenced as a strategy for the spread of this generalist virus [6,27]. Considering that genetic diversity is increased with each CDV transmission from one species to another, it is possible to foresee that the diversity of CDV lineages could vertically reflect the time of presence of the disease in a geographic locality and serve as an indicator of origin and distribution of CDV in different regions [5,6].

Reports of CDV disease in wildlife such as raccoons indicate the causative agent shared a more than 99% identity with sequences obtained from a fox and a domestic dog. This supports interspecies transmission in peri-urban areas [27]. It could be occurring elsewhere, and the impact of CDV on endangered species populations remains to be investigated.

Reports of CDV in wild animals confirm that free-living species interact with domestic canids, but it is not known if pets or wild animals were the source of the infectious agent. The importance of these interactions in transmission has been increasingly demonstrated [28,29]. Limiting the interaction of domestic animals with wildlife could help prevent and control CDV dissemination. These limits could be included in management programs for CDV disease in domestic and agricultural species, and in training programs for personnel involved in studies related to the health and welfare of wildlife in environments close to human populations [4,28].

## 5. Risk as a Potential Zoonotic Disease

Historically, numerous diseases affecting humans caused by viral agents from an animal reservoir have been reported. The genus Morbillivirus includes pathogens that are characterized by high morbidity rates in their various animal reservoir species and in humans, and it is presumed that these infectious agents originate from a common ancestor. The ability of CDV to increasingly expand its host range is now recognized, demonstrating its adaptive capacity [38].

Among morbilliviruses, higher importance is given to MeV, CDV, and Rinderpest Virus (PRV) because they are recognized as agents of important epidemics and epizootics with high mortality rates [2] (Table 2).

The most accepted theory of the common ancestor of these agents is that since the formation of agricultural civilizations, humans maintained constant contact with RPV-infected cattle (reported in epidemics since 376 BC), evolving as MeV and having humans as they only host species (first description of the disease in 900 AC). CDV was first described in America in the early 1700s, years after MeV epidemics occurred on the continent (late 1400s) [2,38].

Based on recent reports, concerns have arisen about possible zoonotic risks of CDV. This is supported by studies in non-human primates, back in 1989 in Japanese macaques (*Macaca fuscata*), and later in 2006 in rhesus monkeys (*Macaca Mulatta*) in China where the presence of measles-like clinical signs was reported. In 2008, this was also noticed in Japan, affecting a population of cynomolgus monkeys [19,20].

Since transmission routes, cellular receptors, their close antigenic relationship, and the clinical manifestations they cause in their hosts are shared, the risk that these animal viruses may affect humans is concerning. Several investigations have focused on studying the similarities between these pathogens and their historical antecedents. These have also led to hypotheses about a possible common ancestor, allowing the approach to discover in which host species the infections originated and raising the possibility of the emergence of pathogens with zoonotic potential [2,38].

Importantly, several events are required for a viral agent to be established in a new species. CDV could have circulated in humans for a long time, and the close contact with canids during domestication could have changed the epidemiology so that the pathogens each adapted to one species with the other becoming immune, thereby limiting interspecies infection and the clinical presentation of disease. This could also be valid for any other related morbillivirus. Thus, MeV vaccination could prevent disease caused by CDV in humans. However, this does not prevent CDV from maintaining the status of a zoonotic risk, and epidemiological changes in MeV or CDV prevention could affect the management of both diseases and the limitation of their species–specific effects.

## 6. Conclusions and Recommendations

CDV is confirmed as widely distributed in species other than canines, affecting terrestrial and aquatic mammals. More studies are needed in Mexico on this disease and its impact on wildlife. In Mexico, there are few reports of wildlife infected with CDV. In aquatic animals, cases of morbillivirus detection have already been reported in sea lions in Baja, California. Coastal areas are inhabited by abundant populations of raccoons that may be susceptible to CDV infection. The presentation of the disease in wild species coincides with what has been studied in domestic canines. Further genetic and pathological studies are recommended to determine the characteristics of these infections and their impact on the affected organisms, as well as to analyze locally the genetic and antigenic variability of CDV with a view on establishing prevention and control tools that include wildlife species.

Mexico is home to at least two wild species that have been reported as reservoirs of CDV infection, based on studies in the Mexico–US interface. Raccoons and coyotes are distributed in several other Mexican states and could play the same reservoir role described for areas in the North American union. The increase in non-conventional species as domestic pets is likely to expand the availability of susceptible hosts to maintain CDV in domestic and peridomestic habitats, contributing to the spread of this disease.

In protected/contained habitats (flora and fauna sanctuaries, reserves, zoological collections, shelters, etc.), populations of wild species and stray dogs (feral canines) mix, potentially spreading CDV infection in both directions. Thus, the circulation of susceptible domestic animals in wildlife habitats should be minimized to avoid forming disease reservoirs.

Vaccination coverage in domestic canids does not reach one hundred percent of the population; this could contribute to maintaining the infection in subclinical conditions, or animals with incomplete vaccination schedules could distribute CDV to other canines or wildlife species in contact. In conservation programs for wildlife species that could be at risk of extinction or populations threatened by widely distributed viral diseases, it is recommended to consider diseases such as the one caused by CDV. Epidemiological surveillance of this disease has predictive value in companion animals and eventually in zoonotic impact.

## Figures and Tables

**Table 1:** Reports of CDV detection in wildlife animals in Mexico. Nomenclature was homologated according to the reference [3].

Common Name (Scientific Name)	Year	Location	Reference
Cozumel Raccoon (*Procyon pygmaeus*)	2007	Cozumel Island, Mexico	[30]
Kinkajú (*Potos flavus*)	2007	Cozumel Island, Mexico	[30]
Wildcat (*Lynx rufus*)	2010	Chihuahua dessert	[31]
Raccoon (*Procyon lotor*)	2015	Monterrey city, Mexico	[1]
Skunk (*Mephitis mephitis*)	2016	Janos, Mexico	[28]
Desert Fox (*Vulpes macrotis*)	2016	Janos, Chihuahua	[28]
Coyote (*Canis latrans*)	2016	Janos, Mexico	[28]
Raccoon (*Procyon lotor*)	2016	Janos, Mexico	[28]
Wildcat (*Lynx rufus*)	2016	Janos, Mexico	[28]
Raccoon (*Procyon lotor*)	2020	Tabasco state, Mexico	[32]
White-nosed Coati (*Nasua narica*)	2020	Tabasco state, Mexico	[32]
Wildcat (*Lynx rufus*)	2021	Janos, Mexico	[29]
Coatí (*Nasua nasua*)	2024	Guadalajara, Jalisco	[29]

**Table 2:** Morbillivirus characteristics, highlighting similarities between MeV and CDV.

Agent (Estimated Time of Appearance)	Transmission	Cellular Receptors	Host Species	Clinical Signs	Current Situation	References
Rinderpest virus (RPV) 376 B.C.	Horizontal	SLAM-CD150 Nectin-4	Order Artiodactyla	Fever Cough Oculo-nasal discharge Conjunctivitis Pneumonia Diarrhea Anorexia Necrotic stomatitis	Eradicated since 2011	[39] [40] [4,38]
Measles Virus (MeV) 900 A.D.	Horizontal. Vertical occasionally	SLAMF1-CD150 Nectin-4 CD46	Human	Fever Rhinorrhea Cough Conjunctivitis Photophobia White spots on fac Skin rashes Diarrhea Encephalitis Ear infection	Effective vaccine control. Significant outbreaks in developing countries.	[41] [4,42,43] [44] [38]
Canine Distemper Virus (CDV) 1735 A.D.	Horizontal. Vertical	SLAM-CD150 Nectin-4 GliaR	Order Families: Carnivorous Artiodactyla Proboscidea Rodentia Pilosa Primates	Fever Cough Nasal discharge Conjunctivitis Diarrhea Skin rashes Plantar hyperkeratosis MyoclonusSeizure Paralysis	Control in domestic dogs. Reports continue in vaccinated animals. Growing range of wildlife hosts.	[2,18,19,40] [4,42,43]
